# Predictors of futile recanalization after intravenous thrombolysis in stroke patients transferred for endovascular treatment

**DOI:** 10.1007/s11239-025-03070-w

**Published:** 2025-02-15

**Authors:** Lucio D’Anna, Matteo Foschi, Luke Dixon, Francesco Bax, Viva Levee, Feras Fayez, Lucinda Knight, Arianna Cella, Alessandro Mare’, Fedra Kuris, Sara Pez, Massimo Sponza, Kyriakos Lobotesis, Thanh Nguyen, Simona Sacco, Gian Luigi Gigli, Mariarosaria Valente, Soma Banerjee, Giovanni Merlino, the Study Investigators*

**Affiliations:** 1 Department of Stroke and Neuroscience, Charing Cross Hospital, Imperial College London NHS Healthcare Trust, London, UK; 2 Department of Brain Sciences, Imperial College London, London, UK; 3 Department of Biotechnological and Applied Clinical Sciences, University of L’Aquila, L’Aquila, Italy; 4 Neuroradiology, Department of Imaging, Charing Cross Hospital, Imperial College London, NHS Healthcare Trust, London, UK; 5 Department of Neurology, Philip Kistler Research Center, Massachusetts General Hospital and Harvard Medical School, Boston, MA USA; 6 Stroke Unit and Clinical Neurology, Udine University Hospital, Udine, Italy; 7 Clinical Neurology, Udine University Hospital and DAME, University of Udine, Udine, Italy; 8 Department of Neurology, Radiology, Boston Medical Center, Boston, MA USA

**Keywords:** Intravenous thrombolysis, Mechanical thrombectomy, Collateral circulation, Futile recanalization, Hub, Spoke

## Abstract

Some patients with large vessel occlusion (LVO) achieve insufficient clinical improvement (futile recanalization, FR) after intravenous thrombolysis (IVT) during inter-hospital transfer for thrombectomy, while others show good outcomes (effective recanalization, ER). This study assessed FR and ER rates among patients treated with IVT at non-thrombectomy primary stroke centers (PSCs) and aimed to identify predictors of FR. We analyzed data from two PSC registries (2016–2022). Inclusion criteria: IVT treatment, anterior circulation LVO, NIHSS ≥ 6, ASPECTS ≥ 5, and documented recanalization at thrombectomy centers. FR was defined as a 90-day poor outcome (mRS 3–6) despite LVO recanalization on initial angiography. Among 190 PSC patients with documented recanalization post-IVT, 113 (59.5%) had FR. Multivariable analysis identified age (OR = 1.03, 95%CI = 1.01–1.07, p = 0.021), NIHSS at the PSC (OR = 1.13, 95%CI = 1.05–1.22, p = 0.026), and collateral status (OR = 0.54, 95%CI = 0.39–0.75, p = 0.001) as independent predictors of FR and 90-day mortality. A model combining age, NIHSS, and collateral score provided the highest predictive accuracy for FR and mortality. FR is common in LVO-related ischemic stroke treated with IVT at non-thrombectomy centers. FR is common in LVO-related ischemic stroke treated with IVT at non-thrombectomy centers. Identifying predictors of FR can guide clinicians in early decision-making, allowing for tailored interventions and informed discussions about expected outcomes, potentially leading to more optimized patient management.

*The GOTIC-VTE trial* Unique identifier, jRCTs031180124; Registration date, April 06, 2017.

**Graphical Abstract:**

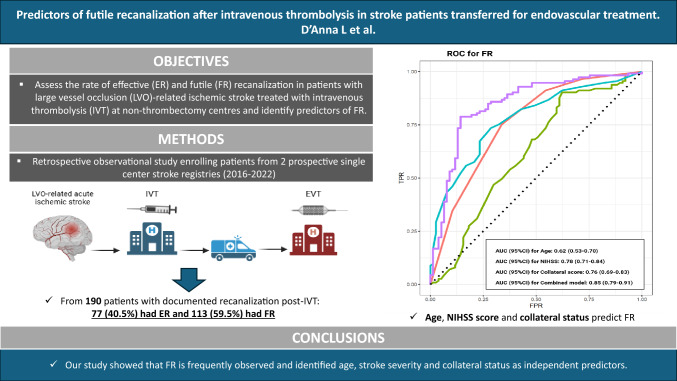

**Supplementary Information:**

The online version contains supplementary material available at 10.1007/s11239-025-03070-w.

## Highlights

- **Futile recanalization (FR) after IVT is a frequent phenomenon**, with 59.5% of patients experiencing poor functional outcomes at 90 days despite documented recanalization. Our study identified **age, NIHSS at the primary stroke center (PSC), and collateral score (CS)** as the key independent predictors of FR. The combination of these variables demonstrated **high predictive accuracy**, with an AUC of 0.85 for FR and 0.86 for 90-day mortality.
- **Collateral circulation plays a crucial role in determining outcomes** after IVT-induced recanalization. Patients with **poor collateral status had a significantly higher risk of FR and mortality**, suggesting that collateral assessment should be an integral part of the initial evaluation to guide clinical decision-making. Interestingly, while treatment times were similar between groups, patients who did not show NIHSS improvement during transfer were more likely to experience FR.
- **A higher risk of hemorrhagic transformation was observed in patients with FR**, emphasizing that the consequences of IVT extend beyond recanalization success. Our findings highlight the importance of early risk stratification to optimize patient selection for EVT and refine expectations regarding prognosis, ultimately leading to **more personalized and effective stroke management strategies**.

## Introduction

Intravenous thrombolysis (IVT) before endovascular treatment (EVT) is the recommended treatment for acute ischemic stroke due to large-vessel occlusion (LVO) [1]. In a ‘hub-and-spoke’ stroke network system, if an LVO is detected at a primary stroke centre (PSC) or spoke centre, patients receive IVT and subsequently are transferred to a comprehensive stroke centre (CSC) or ‘hub’ [2]. However, with this approach some patients ultimately do not undergo EVT because an early recanalization that might occur during the minutes to hours of inter-hospital transfer for EVT. Indeed, despite timely and successful recanalization of the occluded vessel post-IVT, some patients show insufficient clinical improvement. This has been referred to as ‘futile recanalization’ [3, 4] (FR). Previous studies have demonstrated that early treatment, favourable imaging characteristics, thrombus location and composition, age and serum biomarkers are predictors of recanalization in patients with acute ischemic stroke treated with IVT [5–7]. Conversely, delayed treatment, severity of stroke, diabetes and hyperglycaemia are considered predictors of non-recanalization post IVT treatment [5–7]. However, there is a lack of data specifically regarding the rates and the predictors of FR in patients treated with IVT for acute ischemic stroke due to LVO. Identifying patient characteristics associated with poor outcome despite successful IVT-induced recanalization of the occluded vessel may help to improve patient’s management and to set realistic prognostic expectations. Hence, we conducted an analysis from the prospective registries of two thrombectomy capable centres to investigate whether patients with LVO-related ischemic stroke treated with IVT at non-thrombectomy centers can achieve favorable outcomes without requiring EVT. Furthermore, we aim to identify predictors of FR to guide treatment decisions and improve patient prognosis.

## Methods

### Study design, data sources and inclusion criteria

This study combined data from acute stroke patients consecutively admitted to two comprehensive stroke centers for consideration of EVT between January 1st 2016 and December 31st 2022: Charing Cross Hospital, Imperial College Healthcare NHS Trust, London (UK) and Udine University Hospital Santa Maria della Misericordia, Udine (Italy). Local stroke registries are available [8–10]. The Stroke Department at Charing Cross Hospital is a comprehensive stroke center (CSC) and Northwest London (UK) regional lead referral stroke center for EVT in an urban metropolitan area with more than 6.4 million people. It accepts potential candidates 24/7 for EVT from the primary stroke centers (PSC) of the stroke networks that include Luton and Dunstable University Hospital (transfer distance 44 km and transfer time 50 min), Lister Hospital (transfer distance 46 km and transfer time 66 min), Watford General Hospital (transfer distance 29 km and transfer time 45 min), Northwick Park Hospital (transfer distance 9 km and transfer time 28 min), Royal Berkshire Hospital (transfer distance 57 km and transfer time 60 min), High Wycombe Hospital (transfer distance 38 km and transfer time 40 min). The Stroke Department at Udine University Hospital is a CSC serving over 700,000 people in an urban metropolitan in the northeast of Italy. It accepts potential candidates 24/7 for EVT from the PSC at Pordenone Hospital (transfer distance 55 km and transfer time 60 min). The study was conducted in accordance with the recommendations for physicians involved in research on human subjects adopted by the 18th World Medical Assembly, Helsinki 1964 and later revisions. Patients were included in the analysis if they fulfilled the following criteria: (1) age ≥ 18 years; (2) initial admission at a PSC where a standard-of-care Non-Contrast Computed Tomography (NCCT) and Computed Tomography Angiography (CTA) were performed showing an Alberta Stroke Program Early CT score (ASPECTS) [11] 5 or more and an occlusion of the distal internal carotid artery (ICA), the first (M1) or second (M2) segment of the middle cerebral artery; (3) subsequent transfer to a CSC for consideration for EVT; (4) National Institute of Health Stroke Scale (NIHSS) score 6 or more at PSC and on arrival at the CSC; (5) IVT with intravenous tissue plasminogen activator (tPA) administered within 4.5 h of stroke symptom onset and without contraindications according to the guidelines at the PSC; (6) pre-event modified Rankin Scale (mRS) score of 0 to 2; (7) documented recanalization on the first angiography image for the intervention. Recanalization was defined as the dissolution of the thrombus, as confirmed with angiogram performed at the CSC, in the distal internal carotid artery, middle cerebral artery segments M1 or M2 on the first angiography image for the intervention at the CSC by the Interventional Neuroradiologist. Recanalization was assessed by applying the modified thrombolysis in cerebral infarction (TICI) classification [12]. Successful recanalization was defined as grade 2b, 2c or 3 of recanalization.

### Futile recanalization and effective recanalization

We defined futile recanalization (FR) post-IVT as patients experiencing a 90-day poor outcome (mRS 3–6) despite documented recanalization of the LVO on the first angiography image for the intervention at the CSC; effective recanalization (ER) as patients achieving a 90-day good outcome (mRS ≤ 2) with documented recanalization of the LVO on the first angiography image for the intervention at the CSC.

### Data collection

The following variables were collected prospectively: age, sex, vascular risk factors, history of previous stroke or transient ischemic attack (TIA), admission therapy, site of the occlusion, key time points, NIHSS score at the PSC. According to the standard recommendations, the onset to groin puncture time for patients transferred from a PSC to a thrombectomy capable centre should be below 300 min. Improvement of the NIHSS was defined as change in the NIHSS score between presentation to the spoke and presentation to the hub of almost 4 points.

The modified Rankin Scale (mRS) was used to assess the patient’s initial pre-stroke status and the level of functional independence at 90 days of the patients was evaluated centrally through a telemedicine consultation or in-person consultation. The extent of the initial core infarct was determined on pre-therapeutic NCCT using ASPECTS performed at the PSC. NCCT and CTA acquired at the PSC prior to IVT were both available in all the 190 patients. Independent raters (LDX, MS) who did not participate in the endovascular stroke treatment of included patients, evaluated the pre-therapeutic CT to assess the collateral status (CS). CS was based on the 5-point grading system proposed by Souza et al [13]. Intracranial CTA maximum intensity projections were used for the grading the CS: 0 = absent collaterals in > 50% of an MCA M2 branch (superior or inferior division) territory; 1 = diminished collaterals in > 50% of an MCA M2 branch territory; 2 = diminished collaterals in < 50% of an MCA M2 branch territory; 3 = collaterals equal to the contralateral hemisphere; and 4 = increased collaterals (Supplemental Fig. 1).

**Fig. 1:**
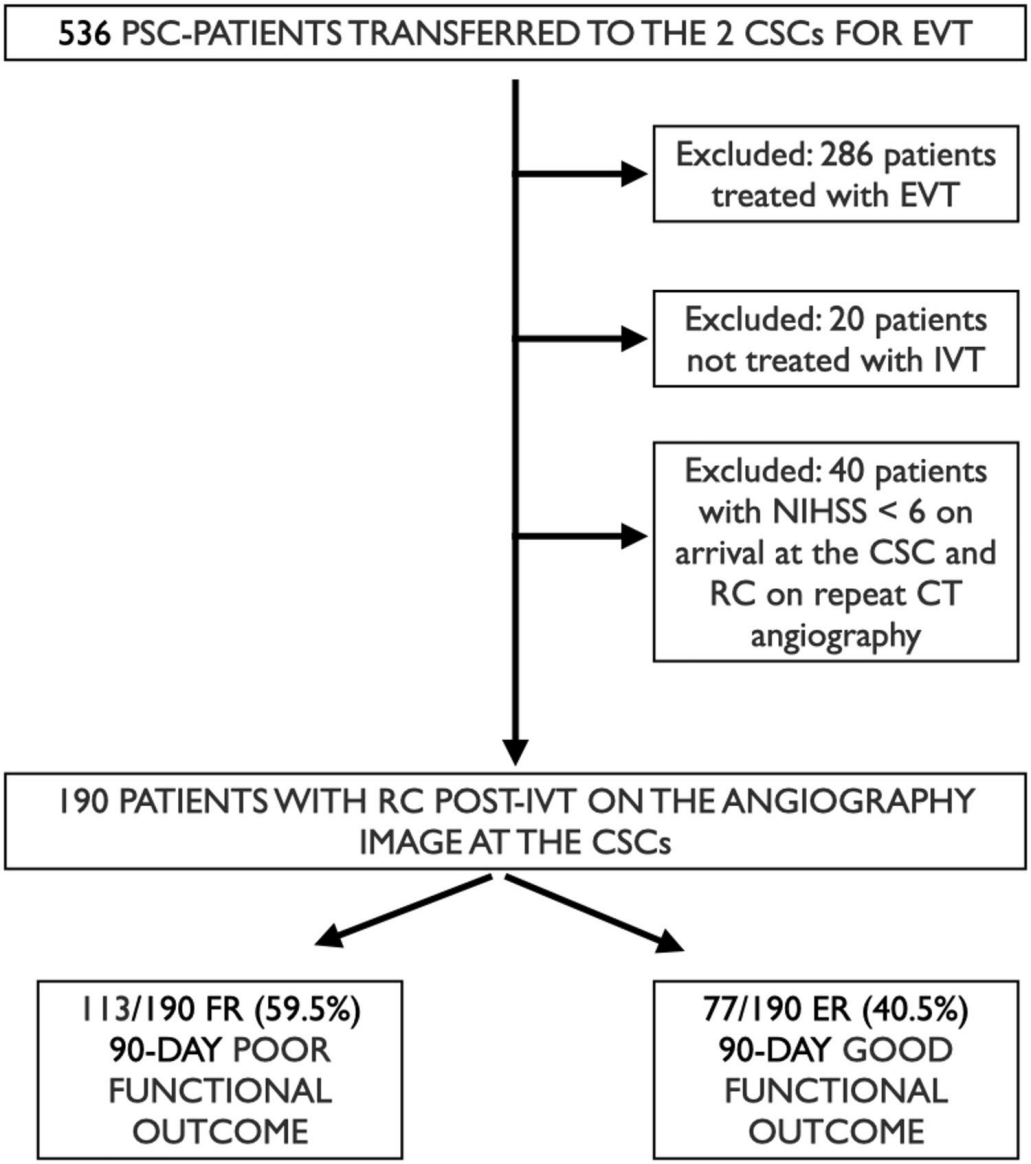
Study algorithm. *EVT* Endovascular treatment, *RC* recanalization, *LVO* large vessel occlusion, *IVT* intravenous thrombolysis, *FR* futile recanalization, *EF* effective recanalization

### Statistical analysis

Statistical analyses were performed with R software, version 4.2.2. Descriptive categorical data were reported as numbers and proportions; descriptive continuous data were reported as means and standard deviations (SDs) for normally distributed variables, or medians and interquartile ranges (IQRs) for non-normally distributed variables. We compared the demographic, clinical, and procedure-related characteristics of patients with FR versus ER using one-way ANOVA (for normally distributed continuous variables, followed by Tukey’s post hoc test), or Kruskal–Wallis test (for non-normally distributed continuous variables). p values were considered statistically significant at < 0.05. We performed a univariate logistic regression analysis with calculation of odds ratios (ORs) and 95% confidence intervals (CIs) to investigate variables associated with the study outcomes. Binary outcomes were defined as follows: favourable (mRS of 0–2) vs. unfavourable (mRS of 3–6) functional outcome at 3-month follow-up; non-survival vs. survival after 90 days; haemorrhagic transformation yes vs. no on follow up CT at 24 h. Variables with a significant association with the study outcomes (p ≤ 0.05) were considered for multivariate logistic regression analysis with statistical significance set at a p < 0.05. The diagnostic values of factors, solely or in combination, to predict FR were tested with ROC curve analysis. For each predictor we provided area under the receiver operating characteristic curves (AUC) with 95%CI in discriminating between patients with 90-day mRS < 3 and 3–6 and between survivors and non-survivors.

## Results

Overall, 536 PSC-patients with anterior circulation LVO transferred to the two CSCs were included. A documented recanalization on the first angiography image for the intervention at the CSC occurred in 190 PSC-patients treated with IVT (Fig. 1, study algorithm). Of these, 77 (40.5%) patients had good functional outcome at 90 days (ER) and 113 (59.5%) had poor functional outcome at 90 days (FR). The clinical characteristics of the two groups of patients are listed in Table 1. ER patients were younger (p = 0.006) and less frequently had diabetes (p = 0.001). We observed a statistically significant distribution of the mRS pre-event between the two groups (p = 0.034), although, as per study inclusion criteria, all the patients had a mRS pre-event of 0 through 2. The NIHSS score at the PSC was significantly higher in the group of patients with FR (p < 0.001), while ER patients more frequently had an improvement of their NIHSS score on arrival at the hub centre (p = 0.004). There were no significant differences between the two groups regarding the key time metrics (Table 1). In supplemental Table 1 the time treatment metrics for each centre are reported.

**Table 1:** Patient characteristics in patients with ER and FR

	Overall population (n=190)	ER (n=77)	FR (n=113)	p value
Age, years [median (IQR)]	73.5 (61.8–80)	68 (54–78)	75 (66–82)	0.006
Female sex [n, (%)]	81 (42.6)	38 (49.4)	43 (38.1)	0.162
Hypertension [n, (%)]	120 (63.2)	43 (56)	77 (68.1)	0.116
KAF [n, (%)]	23 (12.1)	10 (13)	13 (11.5)	0.935
AFDAS [n, (%)]	47 (24.7)	16 (20.8)	31 (27.4)	0.383
Diabetes [n, (%)]	64 (33.7)	11 (14.3)	53 (46.9)	0.001
Hypercholesterolemia [n, (%)]	100 (52.6)	40 (51.9)	60 (53.1)	0.993
Ischemic heart disease [n, (%)]	26 (13.7)	7 (9.1)	19 (16.8)	0.192
Previous IS or TIA [n, (%)]	14 (7.3)	7 (9.1)	7 (6.1)	0.640
mRS pre-event [n, (%)]				0.034
0	127 (66.8)	59 (76.6)	67 (59.3)	
1	38 (20)	9 (11.7)	29 (25.6)	
2	26 (13.7)	9 (11.7)	16 (14.2)	
Antiplatelets [n, (%)]	60 (31.6)	24 (31.2)	36 (31.9)	1
Oral anticoagulant [n, (%)]	3 (1.57)	2 (2.59)	1 (0.88)	0.791
Hb level (g/L), [median (IQR)]	138 (125–146)	137.5 (122.5–146)	138 (129–146)	0.303
Plt count, [median (IQR)]	224 (187–268)	232 (188.5–262.3)	222.5 (181.8–281.3)	0.883
Systolic blood pressure on admission, [median (IQR)]	149 (133.8–164.3)	150 (132.8–164.3)	147 (135.3–163.8)	0.757
Diastolic blood pressure on admission, [median (IQR)]	83 (70–94)	83.5 (74–96)	82 (69.3–93.8)	0.116
PSC-NIHSS, [median (IQR)]	14 (9–18)	10 (6–15)	16 (11–20)	<0.001
Improvement of NIHSS at CSC, [n, (%)]	45 (23.9)	27 (35.1)	18 (15.9)	0.004
Onset to needle time for IVT at PSC, min, [median (IQR)]	127 (92–180)	129 (90–162.3)	125.5 (98.8–185)	0.535
Door to needle time for IVT at PSC, min, [median (IQR)]	39 (27–60)	42 (29–61)	38.5 (26–55)	0.213
Onset to groin puncture time, min, [median (IQR)]	285 (220–344.3)	282.5 (193.8–333.8)	285 (226.3–350)	0.596
Δ Groin puncture time- needle time	158 (120–190)	153 (117–188)	159 (123–191)	0.658
Door to groin puncture time min at CSC, [median (IQR)]	52.9 (33–75)	55.4 (25–80)	52.1 (36–78)	0.094
Door in-door out time at PSC, [median (IQR)]	122.1 (78–161)	119.3 (71–167)	125.3 (67–157)	0.313

*IVT* intravenous thrombolysis, *known atrial fibrillation* KAF, *AFDAS* Atrial fibrillation newly detected in close temporal proximity to the index stroke, *IS* ischemic stroke, *TIA* transient ischemic attack, *NIHSS* National Institute of Health Stroke Scale, *FR* futile recanalization, *ER* effective recanalization

**Fig. 2:**
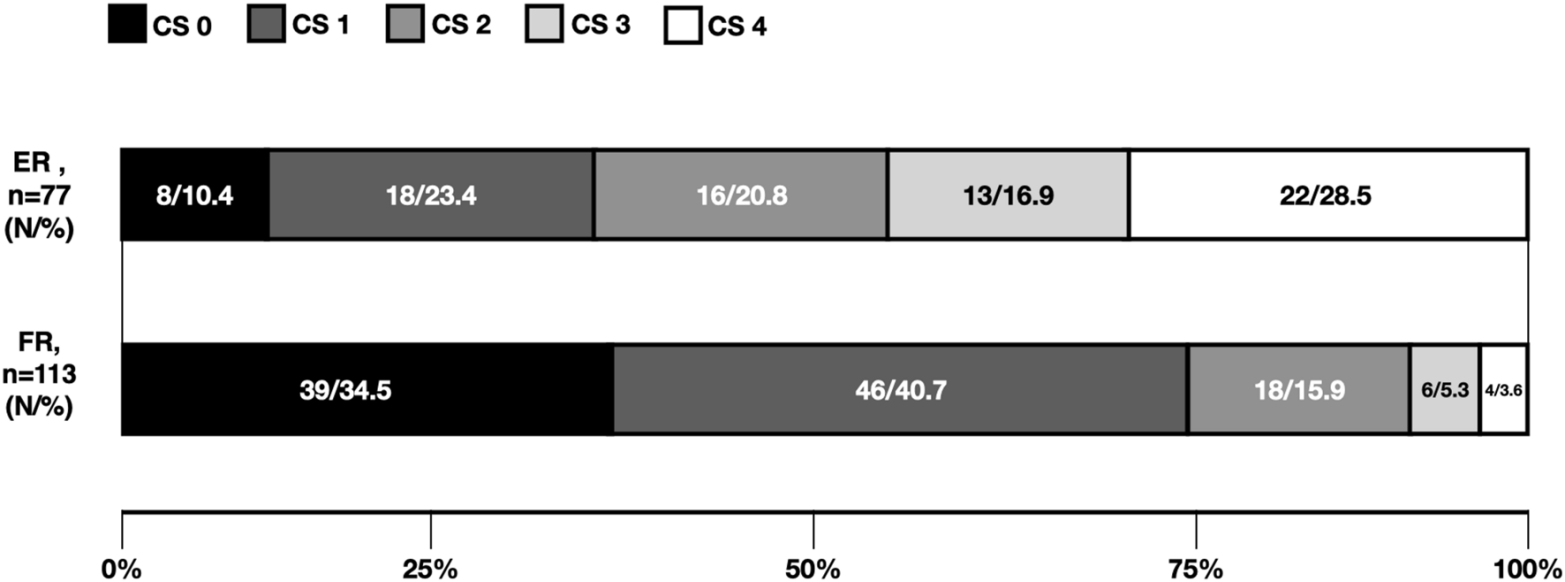
Collateral score distribution. *FR* Futile recanalization, *EF* effective recanalization

In supplemental Table 2 the neuroradiological characteristics of the two groups of patients are reported. FR and ER patients differed significantly in terms of distribution of the CS (p < 0.001) (Fig. 2). However, we did not document statistically significant differences between the two groups of patients in terms of ASPECT score on NCCT and site of the LVO occlusion.

In terms of study outcomes (Table 2), FR patients more often developed haemorrhagic transformation on follow up CT at 24 h compared to ER patients (p = 0.013). Overall, 30 patients out of 190 (15.8%) died at 90 days after the index event. ER patients more often showed an NIHSS improvement of 4 or more points at 24 h (p < 0.001). We did not observe any statistically significant difference in terms of rate of recurrent ischemic stroke within 90 days from the index event between the two groups.

**Table 2:** Outcomes in patients with ER and FR

	Overall population (n=190)	ER (n=77)	FR (n=113)	p value
Haemorrhagic transformation [n, (%)]	32 (16.8)	6 (7.8)	26 (23)	**0.013**
PH, [n, (%)]	13 (6.8)	–	13 (11.5)	–
sICH, [n, (%)]	6 (3.2)	–	6 (5.5)	–
Death at 90 days, [n, (%)]	30 (15.8)	–	30 (15.8)	–
% patients with improvement of NIHSS at 24h*, [n, (%)]	53 (27.9)	40 (51.9)	13 (11.5)	**0.001**
Recurrent ischemic stroke within 90 days from the index event, [n, (%)]	6	2 (2.6)	4 (3.5)	0.698
Death from any other cause	3 (2.6)	–	3 (2.6)	–

*PH* parenchymal haematoma, *sICH* symptomatic intracranial haemorrhage, *FR* futile recanalization, *ER* effective recanalization, *improvement of 4 or more points from the NIHSS score at the CSC

Bold values denote statistical significance at the p < 0.05 level

As shown in Table 3, multivariable regression analysis showed that age per one year (OR = 1.03, 95%CI = 1.01–1.07, p = 0.021), PSC-NIHSS score (OR = 1.13, 95%CI = 1.05–1.22, p = 0.026) and the grade of CS (OR = 0.54, 95%CI = 0.39–0.75, p = 0.001) were independent predictors of FR.

**Table 3:** Univariate and multivariate analysis to predict FR

	Univariate analysis			Multivariate analysis		
	OR (95% CI)	z	p	OR (95% CI)	z	p
Age per one year	1.03 (1.01–1.05)	18.5	0.004	1.03 (1.01–1.07)	10.14	**0.021**
Diabetes	3.55 (1.68–7.46)	28.2	0.001	1.50 (0.57–3.94)	2.28	0.408
mRS pre-event						
0	1			1		
1	2.83 (1.24–6.47)	11.89	0.013	1.69 (0.51–5.65)	2.37	0.387
2	1.56 (0.64–3.80)	2.68	0.322	1.05 (0.29–3.76)	1.08	0.937
PSC-NIHSS, per one point	1.12 (1.07–1.18)	90.6	<0.001	1.13 (1.05–1.22)	26.10	**0.026**
Improvement of NIHSS at CSC	0.35 (0.17–0.70)	0.05	0.003	1.73 (0.32–9.30)	1.90	0.518
Collateral score, per one point	0.44 (0.34–0.58)	0.03	<0.001	0.54 (0.39–0.75)	0.026	**0.001**

*NIHSS* National Institute of Health Stroke Scale, *mRS* modified Rankin scale

Bold values denote statistical significance at the p < 0.05 level

Table 4 shows the logistic regression analysis to determine the predictors of death at 3 months.

**Table 4:** Univariate and multivariate analysis for outcome of death at 90 days post event

	Univariate analysis			Multivariate analysis		
	OR (95% CI)	z	p	OR (95% CI)	z	p
Age per one year	1.07 (1.03–1.10)	29.53	0.001	1.06 (1.01–1.12)	12.83	**0.009**
Diabetes	2.41 (1.09–5.29)	8.99	0.028	1.36 (0.48–3.90)	1.79	0.243
mRS pre-event						
0	1			1		
1	2.82 (1.17–6.78)	10.24	0.020	1.12 (0.31–4.00)	1.19	0.556
2	1.65 (0.54–4.99)	24.31	0.374	8.88 (0.22–3.66)	0.84	0.931
PSC-NIHSS, per one point	1.14 (1.07–1.22)	55.24	<0.001	1.12 (1.02–1.24)	10.43	**0.026**
Improvement of NIHSS at CSC	0.17 (0.04–0.78)	0.10	0.026	0.33 (0.02–6.05)	0.48	0.649
Collateral score, per one point	0.34 (0.20–0.56)	0.02	<0.001	0.44 (0.24–0.81)	0.07	**0.001**

*NIHSS* National Institute of Health Stroke Scale; *mRS* modified Rankin scale

Bold values denote statistical significance at the p < 0.05 level

Multivariable regression analysis showed that age per one year (OR = 1.06, 95%CI = 1.01–1.12, p = 0.009), PSC-NIHSS score (OR = 1.12, 95%CI = 1.02–1.24, p = 0.026) were independent predictors of death at 3 months after the index event. We also found that the CS (OR = 0.44, 95%CI = 0.24–0.81, p = 0.001) was inversely correlated with the risk of death at 3 months.

Supplemental Table 3 shows the logistic regression analysis to determine the predictors of haemorrhagic transformation on follow up CT at 24 h. Multivariable regression analysis showed that the PSC-NIHSS score (OR = 1.10, 95%CI = 1.04–1.16, p < 0.001) was an independent predictor of haemorrhagic transformation.

**Fig. 3:**
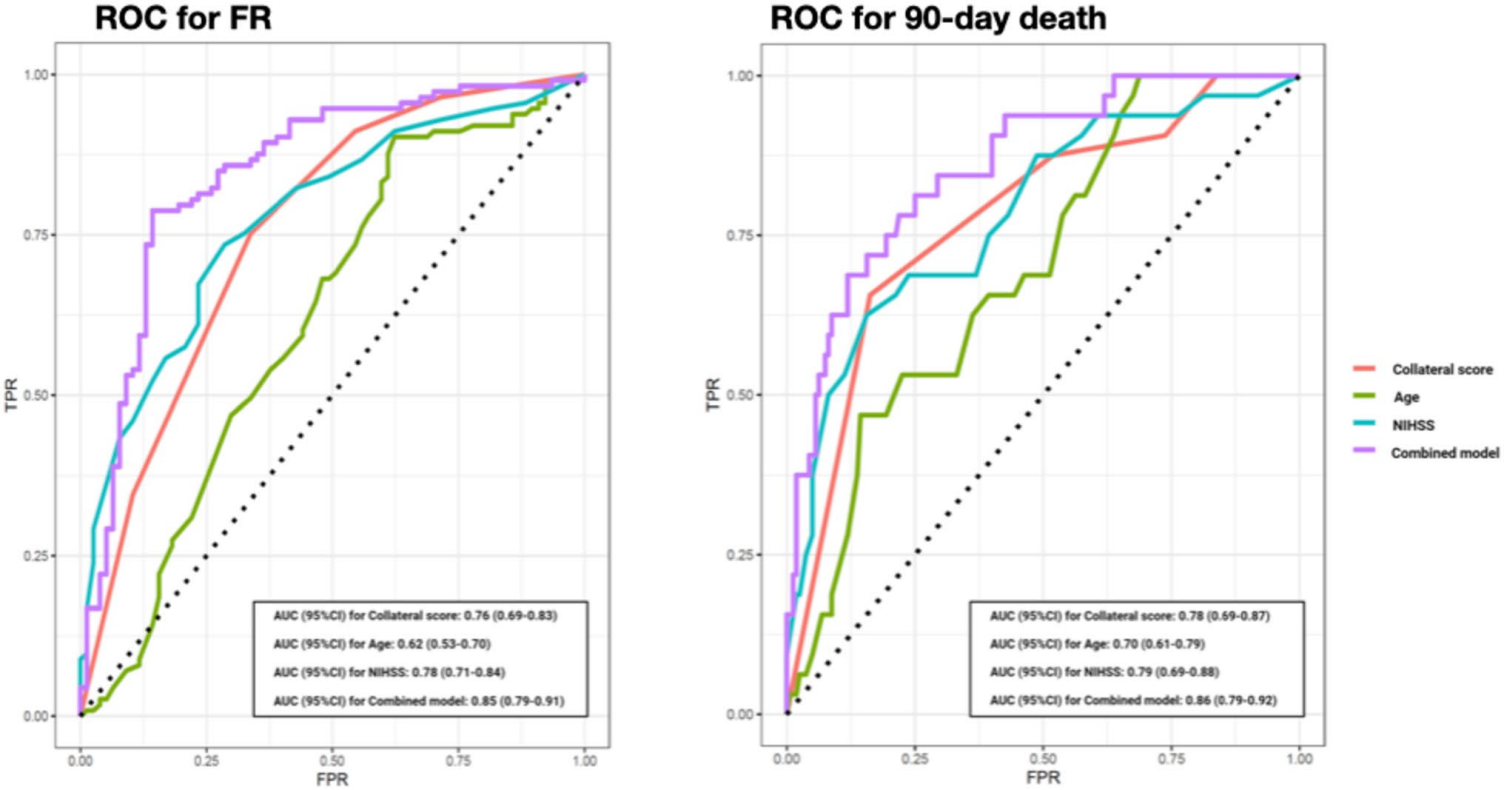
ROC curves to predict futile recanalization and death at 90-days

Using the ROC curves from the logistic regression analysis, we identified the predictive accuracy of age, CS and NIHSS score at the PSC for FR and death (Fig. 3). The combined model including CS, age and NIHSS at the PSC showed the highest prognostic performance for both risk of FR and death (AUC [95%CI]: 0.85 [0.79–0.91] and 0.86 [0.79–0.92], respectively.

## Discussion

Our study of patients with LVO-related anterior circulation stroke transferred from a PSC to CSC for consideration of thrombectomy showed that: (1) 59.5% of PSC patients treated with IVT experience poor 90-day functional outcome despite documented recanalization; (2) baseline variables obtained at the PSC that are independently associated with risk of FR and 90-day death include age, PSC-NIHSS score and CS.

The rates of recanalization following IVT treatment show considerable variation across various studies. Flores et al. [14] showed that partial or complete recanalization occurred in 10.5% of patients with a confirmed LVO in the PSC transferred to a CSC for EVT evaluation while Ospel et al. documented 41% recanalization rate [15]. However, the rates and predictors of FR in these patients remain uncertain. Recanalization is the mainstay of ischemic stroke treatment and is generally considered a prognostic factor for good outcome [16, 17]. Recanalization is the prerequisite to establish reperfusion. Several processes including distal clot fragmentation, pericyte constriction or poor collaterals may hamper reperfusion of the ischemic brain [18]. Therefore, progressive infarct expansion in patients with poor clinical outcome despite recanalization is caused by reperfusion failure. In our study we showed that patients with FR compared to those with ER had a statistically significant difference in terms of the distribution of the CS. Indeed, FR patients more often presented lower collateral grades compared to their counterparts with ER_._ Of note, the two groups of patients did not have significant differences in terms of any median treatment time delays taken into consideration, ASPECTS on NCCT and site of the LVO occlusions. Our data are in line with the results of a recent landmark trials of endovascular stroke therapy [19] showing that at least 50% of patients with anterior circulation LVO may be ‘fast progressors’, whose infarct growth is most sensitive to duration of ischemia because of rapid failing collaterals and who would benefit from fastest possible access to EVT within the early time window [20].

In our analysis CS, obtained with the CTA at the PSCs, correlated with 90-day functional outcome and risk of death. Collateral circulation of the brain refers to alternative vessels, consisting of primary circle of Willis and secondary pial leptomeningeal anastomoses, that can compensate for reduced blood flow in the setting of LVO [21]. Indeed, after arterial occlusion, there can be temporal growth of the ischemic core into the penumbral area that is modulated by collateral blood flow, the key element setting the pace of the ischemic process [22, 23]. Collateral patterns assessed by presentation CTA vary dramatically among patients with stroke and are highly related to larger volumes of salvageable ischemic tissue, slow rate of ASPECTS decay between hospital transfer and improved clinical outcomes [24–26]. A strong body of evidence has demonstrated that collateral status plays a crucial role in the prognosis of patients with acute ischemic stroke. Leng et al. demonstrated the prognostic value of baseline collateral circulation for outcomes of acute ischemic stroke patients receiving IVT [27]. However, the authors did not specify the percentage of patients with acute ischemic stroke due to anterior circulation LVO. Conversely, a previous meta-analysis of twelve studies enrolling 2138 patients with acute ischemic stroke due to anterior circulation LVO, treated with or without IVT, failed a significant impact of collateral status on futile recanalization after MT treatment [28]. This was mainly due to the limited number of studies, variation in scales assessing collateral status, and presence of heterogeneity. Overall, the latest American Heart Association/American Stroke Association guidelines suggest that it may be reasonable to incorporate collateral flow status into clinical decision-making in some candidates to determine eligibility for MT with a IIb level of evidence although no specific criteria are suggested [1].

In our study we documented also that age and PSC-NIHSS score independently predicting functional outcome after IVT-induced recanalization. We also found that grade of CS in combination with age and severity of stroke syndrome represented a model with good predictive accuracy for poor outcome and death at 3 months after the index. Several models have been developed to predict poor functional outcome despite recanalization of the LVO in patients treated with EVT rather than for IVT-only patients [28–35]. The MR PREDICT tool combing multiple baseline clinical and radiological characteristics aided in distinguishing between patients who may experience benefit from intra-arterial treatment for acute ischemic stroke and those will not [36]. Meinel et al. developed and validated a multivariable prognostic model to prospectively predict futile recanalization therapies in patients with acute ischemic stroke [37]. The authors documented that several clinical variables (higher stroke severity, older age, active cancer, pre-stroke disability), laboratory values (higher glucose, higher C-reactive protein), imaging biomarkers (more white matter hyperintensities), and longer onset-to-admission time were associated with futile recanalization. However, only 42.6% of the patients included in their analysis had a detectable LVO and only 11% of the patients received IVT alone. Our model might be able to identify the most relevant features in the emergency setting that can predict outcomes in these patients with early recanalization of the LVO post-IVT.

Finally, our data showed that patients who experienced FR were more likely to develop haemorrhagic transformation on follow-up CT at 24 h. Haemorrhagic transformation is a known complication of IVT. In our study, the higher incidence of haemorrhagic transformation in the futile recanalization group suggests that these patients may be at greater risk of adverse outcomes not solely due to the recanalization status but also due to the underlying pathology and treatment complications. This highlights the need to consider both factors in evaluating patient outcomes. This observation warrants further investigation to differentiate the direct effects of recanalization efficacy from those of therapy-related complications.

Our study has the following strengths: (1) large cohort of patients; (2) recanalization was assessed with angiography image for intervention at the two thrombectomy capable centres. Nevertheless, our study has several limitations. First, the observational design of the study. We did not routinely repeat a NCCT in spoke patients with acute ischemic stroke on arrival at the CSC when patients presented an NIHSS > 6 and per our criteria were eligible for EVT. Moreover, our study provides insights into the predictors of futile recanalization, these predictors primarily aid in post-transfer processes, such as prognostication and care planning, rather than influencing the initial decision to transfer a patient for thrombectomy. The immediate clinical decision to transfer should continue to prioritize timely and effective treatment for AIS due to LVO.

In conclusion, our study showed the rates and predictors of FR in patients with acute ischemic stroke due to LVO transferred from a PSC to a CSC. Our findings also highlight the importance of assessing collateral circulation as part of the routine neuroimaging protocols for PSC-patients with acute ischemic stroke when considering a transfer to a CSC for intervention. In the emergency setting, identifying predictors of FR can guide clinicians in early decision-making, allowing for tailored interventions and informed discussions about expected outcomes, potentially leading to more optimized patient management.

## Supplementary Information

Below is the link to the electronic supplementary material.

Supplementary file1 (JPEG 647 KB)

Supplementary file2 (DOCX 21 KB)

## Data Availability

The data that support the findings of this study are available on request from the corresponding author. The data are not publicly available due to privacy or ethical restrictions.
