# Supplementary figures and images for: Predictors of futile recanalization after intravenous thrombolysis in stroke patients transferred for endovascular treatment
Source: J Thromb Thrombolysis. 2025 Feb 15;58(2):232–42. doi: 10.1007/s11239-025-03070-w (PMC11885372; doi:10.1007/s11239-025-03070-w)

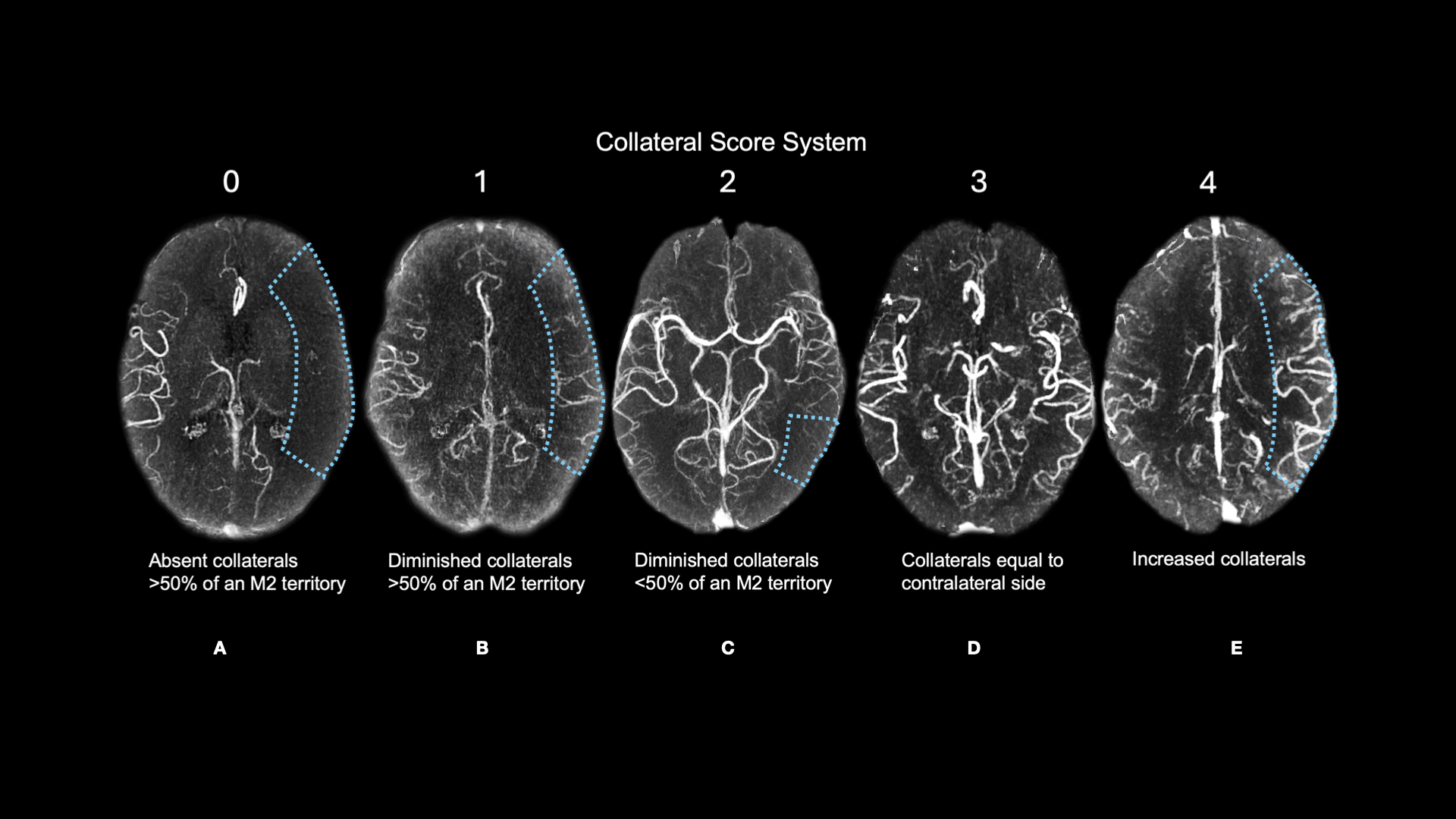

Supplement: Supplementary file 1 — Supplementary file1 (JPEG 647 KB) [file 11239_2025_3070_MOESM1_ESM.jpeg]
