# Supplementary material for: Predictors of futile recanalization after intravenous thrombolysis in stroke patients transferred for endovascular treatment
Source: J Thromb Thrombolysis. 2025 Feb 15;58(2):232–42. doi: 10.1007/s11239-025-03070-w (PMC11885372; doi:10.1007/s11239-025-03070-w)
Supplement: Supplementary file 2 — Supplementary file2 (DOCX 21 KB) [file 11239_2025_3070_MOESM2_ESM.docx]

**Supplemental Table 1. Treatment time at each hospital.**

|  | Charing Cross Hospital, Imperial College Healthcare NHS Trust | Udine University Hospital Santa Maria della Misericordia | Charing Cross Hospital, Imperial College Healthcare NHS Trust | Udine University Hospital Santa Maria della Misericordia |
| --- | --- | --- | --- | --- |
|  | ER | | FR | |
| Onset to needle time for IVT at PSC, min, [median (IQR)] | 128  (89-162) | 130  (91-161) | 125 (99-185) | 125 (98-185) |
| Door to needle time for IVT at PSC, min, [median (IQR)] | 41 (29-62) | 43 (28-59) | 39 (27-55) | 38 (26-55) |
| Onset to groin puncture time, min, [median (IQR)] | 281 (194-334) | 281.5 (193-335) | 284 (226-351) | 283 (225-349) |
| Δ Groin puncture time- needle time | 151 (116-188) | 152 (117-189) | 158 (122-192) | 157 (121-190) |
| Door to groin puncture time min at CSC, [median (IQR)] | 56 (24-79) | 54 (26-81) | 51.9 (37-78) | 52 (36-79) |
| Door in-door out time at PSC, [median (IQR)] | 120 (70-166) | 118 (70-168) | 124 (68-156) | 126 (66-157) |

Futile recanalization (FR); effective recanalization (ER).

**Supplemental Table 2. Neuroradiological characteristics of ER and FR patients.**

|  | **Overall population (n=190)** | **ER**  **(n=77)** | **FR**  **(n=113)** | **P value** |
| --- | --- | --- | --- | --- |
| Collateral score |  |  |  | **<0.001** |
| 0 [n, (%)] | 47 (24.7) | 8 (10.4) | 39 (34.5) |  |
| 1 [n, (%)] | 64 (33.7) | 18 (23.4) | 46 (40.7) |  |
| 2 [n, (%)] | 34 (17.9) | 16 (20.8) | 18 (15.9) |  |
| 3 [n, (%)] | 19 (10) | 13 (16.9) | 6 (5.3) |  |
| 4 [n, (%)] | 26 (13.7) | 22 (28.5) | 4 (3.6) |  |
| ASPECTS, [median (IQR)] | 8 (8-10) | 9 (8-10) | 8 (7-10) | 0.064 |
| Site of the occlusion [n, (%)] |  |  |  | 0.588 |
| Distal ICA | 10 (5.3) | 2 (2.6) | 8 (7.1) |  |
| M1 | 110 (57.9) | 47 (61.0) | 63 (55.8) |  |
| M2 | 54 (28.4) | 23 (29.9) | 31 (27.4) |  |
| Tandem occlusion ICA+M1 | 16 (8.4) | 5 (6.5) | 11 (9.7) |  |

Legend: Primary Stroke Centres (PSC); Comprehensive Stroke Centres (CSC); intravenous thrombolysis (IVT); recanalization (RC); RC with good functional outcome (RC_GO_); RC with poor functional outcome at 90 days (RC_PO_); IVT administered at PSC (+PSC-IVT); Alberta Stroke Program Early CT score (ASPECTS); internal carotid artery (ICA).

**Supplemental Table 3. Univariate and multivariate analysis for outcome of haemorrhagic transformation post event.**

|  | **Univariate analysis** | | | **Multivariate analysis** | | |
| --- | --- | --- | --- | --- | --- | --- |
|  | **OR (95% CI)** | **z** | **p** | **OR (95% CI)** | **z** | **p** |
| Age per one year | 1.02 (0.98-1.05) | 2.76 | 0.301 |  |  |  |
| Diabetes | 2.00 (0.90-4.41) | 5.58 | 0.086 |  |  |  |
| mRS pre-event |  |  |  |  |  |  |
| 0 | 1 |  |  |  |  |  |
| 1 | 2.27 (0.94-5.49) | 6.14 | 0.070 |  |  |  |
| 2 | 1.51 (0.50-4.54) | 2.08 | 0.461 |  |  |  |
| PSC-NIHSS, per one point | 1.10 (1.04-1.16) | 35.71 | <0.001 |  |  |  |
| Improvement of NIHSS at CSC | 1.35 (0.57-3.18) | 1.99 | 0.489 |  |  |  |
| Collateral score, per one point | 0.99 (0.1-1.10) | 1.09 | 0.275 |  |  |  |

Legend: National Institute of Health Stroke Scale (NIHSS); modified Rankin scale (mRS).
